# Effects of Difenoconazole on *Tubifex tubifex*: Antioxidant Activity, Insights from GUTS Predictions, and Multi-Biomarker Analysis

**DOI:** 10.3390/biology14030302

**Published:** 2025-03-17

**Authors:** Subhajit Saha, Shubhajit Saha, Paolo Pastorino, Nimai Chandra Saha

**Affiliations:** 1 Department of Zoology, West Bengal State University, North 24 Paraganas, Barasat 700126, West Bengal, India; subhajitsaha960@gmail.com; 2 Fisheries and Ecotoxicology Research Laboratory, Department of Zoology, The University of Burdwan, Burdwan 713104, West Bengal, India; ssaha@zoo.buruniv.ac.in; 3 Istituto Zooprofilattico Sperimentale del Piemonte, Liguria e Valle d’Aosta, 10154 Torino, Italy; 4 Department of Zoology, Bidhannagar College, Bidhannagar, Kolkata 700064, West Bengal, India

**Keywords:** acute toxicity, fungicide, non-target organisms, oxidative stress, sublethal exposure

## Abstract

The increasing demand for agricultural products has led to a rise in pesticide use, resulting in the pollution of aquatic habitats and raising significant health concerns for both aquatic life and humans. Difenoconazole, a triazole fungicide, is becoming increasingly popular in agriculture, yet its effects on non-target organisms, such as annelids, are not well understood. This study aimed to investigate the toxicological effects of difenoconazole and assess its potential impact on toxicity biomarkers, using *Tubifex tubifex* as a model organism, to better understand the ecotoxicity of difenoconazole on freshwater annelids. The 96-h LC_50_ value of difenoconazole was determined to be 2.68 mg/L. Sublethal concentrations (10% and 20% of the 96-h LC_50_ value; 0.268 and 0.536 mg/L, respectively) caused significant changes in the activities of oxidative stress enzymes. A concentration- and time-dependent decrease in the activities of superoxide dismutase (SOD), catalase (CAT), and glutathione transferase (GST) was observed compared to control organisms. Additionally, malondialdehyde (MDA) concentrations increased throughout the exposure period. An Integrated Biomarker Response (IBR) assessment was used to characterize and illustrate the impact of difenoconazole on *T. tubifex*. In conclusion, exposure to this fungicide appears to reduce the survival rate of *T. tubifex* at acute levels and disrupt its normal behavioral patterns. Moreover, it alters oxidative stress enzyme levels during sublethal exposure. Long-term exposure to the fungicide could potentially have population-level consequences, including a reduction in the number of individuals within a population.

## Simple Summary

The widespread use of pesticides in agriculture can pollute waterbodies, posing risks to aquatic life and human health. Difenoconazole, a commonly used fungicide, may affect freshwater organisms like *Tubifex tubifex*, but its impact is not well understood. This study investigated how difenoconazole influences the health of these worms by analyzing changes in their antioxidant enzyme activity. The results showed that even low concentrations of the pesticide reduced the worms’ ability to cope with environmental stress, potentially affecting their survival and behavior. Higher concentrations were found to be lethal within a short period. These findings suggest that long-term exposure to difenoconazole could lead to a decline in worm populations, which are important for maintaining aquatic ecosystem balance. Understanding these effects can contribute to improved environmental protection measures and sustainable agricultural practices.

## 1. Introduction

Agriculture commonly employs pesticides to enhance crop yields [1,2]. The use of fungicides as both preventive and curative agents against fungal diseases is widespread globally [3]. However, the extensive and improper application of fungicides can have detrimental effects on both animals and plants [4]. Fungicides significantly reduce the richness and abundance of terrestrial and aquatic animals, as well as microbial life. They also contaminate drinking water sources, which are critical for human well-being [5]. Pesticides can infiltrate various food chains, including those involving humans. These chemicals can accumulate in the bodies of aquatic and terrestrial animals, eventually reaching humans and causing harmful effects [6,7,8,9]. Therefore, the use of these substances should be regulated based on scientific principles to minimize potential harm to health and the environment [10].

Fungicides containing the azole group are widely used in agriculture, veterinary medicine, and human medicine as broad-spectrum antifungal agents [11,12]. Based on their structural characteristics, azoles are classified into imidazoles (with two nitrogen atoms) and triazoles (with three nitrogen atoms) [13]. Triazole fungicides are among the most widely used pesticides in agriculture due to their broad spectrum, long-lasting effects, and high efficiency [14]. Azole substances account for approximately 20–25% of the global fungicide market’s value, largely driven by their role in crop protection [15]. However, this extensive use has led to the accumulation of toxic residues in surface waters and everyday environments, harming both ecosystems and animal health [16].

Difenoconazole, a triazole fungicide, currently holds a significant share of the global pesticide market [17]. It is commonly applied to various crops, including broccoli, winter wheat, rapeseed, cabbage, cauliflower, and Brussels sprouts [18]. This fungicide inhibits fungal growth by inactivating the cytochrome P450 enzyme (CYP51), which is involved in ergosterol biosynthesis [3]. Difenoconazole is highly persistent in water and soil due to its chemical stability, poor biodegradability, and ease of transfer [17]. Consequently, it has been detected in agricultural water and surface layers of water worldwide. For example, the concentrations of difenoconazole have reached up to 0.028 mg/L in agricultural water in Thailand [19], 1.98−2.91 mg/L in paddy water in China [20], and 0.30 mg/L in surface water in Malaysia [21], with a value of 0.15 µg/L also recorded [22].

Short-term exposure to difenoconazole appears to be more harmful to aquatic species than other triazole fungicides [23]. While numerous studies have assessed the toxicity and safety of difenoconazole in various animals, these have mostly involved standard model organisms, such as fish, daphnia, algae, and bees [24,25,26,27,28]. Difenoconazole is highly toxic to the aquatic species *Daphnia magna*, causing severe chronic toxicity with a NOEC of 0.0056 mg/L [29]. This substance has also been found to inhibit brain and ovarian aromatase activities in rainbow trout [30]. It affects various behavioral and physiological endpoints, as well as development and gene expression, in both adult and larval zebrafish (*Danio rerio*) [31]. Additionally, difenoconazole has been shown to damage the antioxidant system, promote apoptosis in carp spleen tissue, and cause cardiotoxicity [17,32]. Recent research by Dornelas et al. [33] suggests that difenoconazole reduces the fertility and fecundity of *Girardia tigrina*, a non-target aquatic organism. Despite toxicological studies on difenoconazole involving many phyla, data on its effects on annelids remain scarce. Therefore, further environmental toxicological studies are needed to assess the risks posed by difenoconazole to aquatic organisms.

A variety of biomarkers or early indicators, such as behavioral changes, oxidative stress, and histoarchitectural alterations, can be used to evaluate the health of aquatic organisms [34,35,36,37,38]. To combat free radicals, animals produce antioxidant enzymes such as superoxide dismutase (SOD), catalase (CAT), and glutathione transferases (GSTs) [39,40]. Changes in these antioxidant enzymes are typically viewed as potential indicators of increase reactive oxygen production (ROS) production. Moreover, excessive ROS production can damage various cellular components, including proteins, DNA, and lipids, potentially altering the histopathology of the affected tissues [41].

In this study, we have selected the freshwater oligochaete worm *Tubifex tubifex* as our model organism. This species is highly tolerant of toxic and polluted conditions, as well as low oxygen levels, making it ideal for studying the effects of water and sediment contamination. Additionally, it is easy to breed in laboratory conditions. Therefore, the present investigation aims to evaluate the toxicological impact of difenoconazole on *Tubifex tubifex* across various behavioral and physiological endpoints. The mortality rate of *Tubifex tubifex* was assessed using an acute toxicity test with difenoconazole. The worms’ behavioral responses and the percentage of autotomy were observed at different concentrations of the fungicide. Histological architecture, as well as the activities of several antioxidant enzymes (CAT, SOD, and GST) and levels of oxidative damage of lipids (MDA) were assessed after sub-acute exposure to the fungicide. IBR analysis was performed to evaluate the efficacy of stress enzymes in *Tubifex tubifex* exposed to sublethal concentrations of difenoconazole.

## 2. Material and Methods

### 2.1. Chemicals

The commercially available fungicide difenoconazole (Syngenta Score, 25% EC) and other chemicals and reagents were obtained from Sisco Research Laboratories Pvt. Ltd. (SRL), Mumbai, India, and HiMedia Laboratories Pvt. Ltd., Mumbai, India.

### 2.2. Test Animal

Mature *Tubifex tubifex*, a species from the order Tubificidae, were purchased from a local market in Bardhaman, West Bengal, India. The worms were reacclimated to laboratory conditions by placing them in a plastic tray with clean, unchlorinated running tap water for 24 h. Only healthy and physically active organisms with an average length of 3.05 ± 0.07 cm were selected. After acclimation, they were transferred to 250 mL glass beakers containing 200 mL of tap water. The water used for the experimental bioassay was maintained at the following physicochemical parameters: temperature = 20.6 ± 1.67 °C, pH = 7.35 ± 0.4, free CO_2_ = 17.9 ± 1.34 mg/L, dissolved oxygen = 6.64 ± 0.37 mg/L, total alkalinity = 188.4 ± 2.16 mg/L as CaCO_3_, and hardness = 130.4 ± 1.87 mg/L as CaCO_3_.

### 2.3. QA/QC (Quality Assurance/Quality Control) Procedure

The extraction and desorption conditions were determined based on previous studies [42,43]. The extraction process used 100 mL of the sample. Menezes et al. [42] evaluated LPME (Liquid-Phase Microextraction) samples in two and three phases, including variables such as salt addition, stirring speed, and extraction time. A 6.0 cm long hollow polypropylene fiber with an internal diameter of 600 μm and a wall thickness of 200 μm was used for extractions. All studies were conducted in triplicate. The investigation was carried out using a Shimadzu GC/MS system (model GC−2010/QP−2010) from Kyoto, Japan. A capillary column (30 m × 0.25 mm × 0.25 μm) with 5% diphenyl and 95% dimethylpolysiloxane (HP-5MS) from Agilent Technology Inc. (Santa Clara, CA, USA) was utilized. Nominal concentrations were used throughout the experiment, as the difference between the nominal and observed values was less than 5% (Table S1; Figure S1). After every five batches of sample analysis, standard reference materials were used to detect deviations from calibration standards and to eliminate equipment drift [44]. All samples were tested multiple times to ensure precision and accuracy, with a detection limit of 0.006 mg/L determined through the analysis of spiked samples.

### 2.4. Median Lethal Concentration Determination

Acute toxicity tests were conducted in 250 mL glass beakers containing 200 mL of water, with 10 worms in each beaker. For greater statistical validity, each experiment was conducted in triplicate. Initially, a range-finding test was used to determine the concentration range at which mortality occurred. *Tubifex tubifex* was then exposed to various nominal concentrations of the fungicide difenoconazole (2.00, 2.50, 3.00, 3.50, 4.00, 4.50, 5.00, 5.50, 6.00, and 6.50 mg/L) along with a non-exposed control group. During the 96 h acute toxicity bioassay, 10% of the medium was replaced daily, with the corresponding amount of difenoconazole added to maintain the desired toxicant concentration in the closed environment. The worms were observed every 24 h over the 96 h period to assess mortality and survivability. Mortality was confirmed when *T. tubifex* stopped responding even after being touched with a clean pointed paintbrush. The median lethal concentration (LC_50_) of difenoconazole for *T. tubifex* was determined using Finney’s Probit statistical tool [45]. Additionally, the Kaplan–Meier survival plot was used to demonstrate the adverse effects of difenoconazole on the overall survivability of the worms.

### 2.5. Behavioral Observation

A semi-quantitative scoring technique, as described by Dhara et al. [46], was used to examine the physiological and behavioral changes induced by toxicants. Observations were made at 24 h intervals during the 96 h exposure period, noting behavioral indicators such as mucus release, hyperactivity, clumping tendency, and wrinkling.

### 2.6. Oxidative Stress Biomarkers

Two sublethal concentrations of difenoconazole (10% and 20% of the 96-h LC_50_ value, i.e., 0.268 mg/L and 0.536 mg/L, respectively) were used to study the oxidative stress enzyme levels. *Tubifex tubifex*, weighing approximately 5 g, were transferred from the stock tray to experimental aquariums containing 3 L of chlorine-free tap water. Control worms were placed in a separate aquarium with 3 L of toxin-free tap water. These experimental setups were performed in triplicate, with 10% of the test medium replaced every 2–3 days. On days 1, 7, and 14, 1.0 g of *Tubifex* worms from the control and difenoconazole-treated aquariums were removed, homogenized in phosphate buffer, and centrifuged at 10,000 rpm for 15 min at 4 °C; the supernatant was stored at −20 °C until enzymatic analysis. Protein concentrations of each sample were determined using the Bradford (1976) method.

The activities of superoxide dismutase (SOD), catalase (CAT), and glutathione S-transferase (GST) and the concentration of malondialdehyde (MDA) were estimated according to published protocols [47,48,49,50]. SOD, CAT, and GST activities were expressed in units of U/mg protein, while MDA activity was expressed in nmol TBARS/min/mg protein.

### 2.7. Integrated Biomarker Response (IBR)

The Integrated Biomarker Response (IBR) is a model that synthesizes multiple biomarkers to better understand environmental health risks. This model is easily integrated into environmental control frameworks [9,34,51,52]. The IBR index provides a comprehensive measure of the involvement of stress factors in the physiological changes of fish and other aquatic organisms [53]. Oxidative stress biomarkers were measured and integrated using IBR, and the responses of *T. tubifex* to difenoconazole were evaluated using radar plots [54].

### 2.8. Histological Alterations

Histopathological analysis was conducted after 14 days of exposure. Worms from the control, T1, and T2 treatment groups (n = 60, 10 worms per replicate) were sacrificed, and their tissues were collected. The tissues were fixed in 4% formalin for 48 h, dehydrated in graded alcohols, cleared with xylene, and embedded in paraffin wax. Sections 5 μm thick were cut using an automated microtome (RM-2155, Leica, Vienna, Austria), stained with hematoxylin and eosin (H&E), and mounted in DPX. The slides were examined using a binocular research microscope connected to a digital camera (model: DIGI510, Dewinter, New Delhi, India, with a 5.1 MP camera, 1/2.5″ Aptina CMOS sensor).

### 2.9. Statistical Analysis

The LC_50_ values and their fiducial intervals were calculated using IBM SPSS Statistics (v. 26) software with probit analysis based on the mean mortality of *Tubifex tubifex* after 24, 48, 72, and 96 h of exposure [55]. Data analysis was conducted using GraphPad Prism (version 9.5.0) and R Studio (version 1.4.1564), and diagrams were created accordingly. Kaplan–Meier analysis was used to predict the survival of the test organisms during exposure. The General Unified Threshold model of Survival (GUTS) was applied using the standalone open-source OpenGUTS program, providing evidence to support the survivability prediction. This analysis helped determine the mechanisms of action of difenoconazole on *Tubifex tubifex*. Two-way ANOVA and Tukey’s post hoc analysis were employed to identify differences between the control and treated groups. Statistical significance was considered at levels of *p* < 0.05. The toxicity factor (TF) of this chemical was calculated according to the formula developed by [56] based on different exposure periods (24, 48, 72, and 96 h):

$$Toxicity\;factor\;\left(TF\right)=\frac{{LC}_{50}\;value\;at\;24\;h}{{LC}_{50}\;value\;at\;any\;other\;exposure\;period}$$

## 3. Results and Discussion

### 3.1. Acute Toxicity Assessments (96 h)

#### 3.1.1. Determination of the Lethal Concentrations

**Figure 1:**
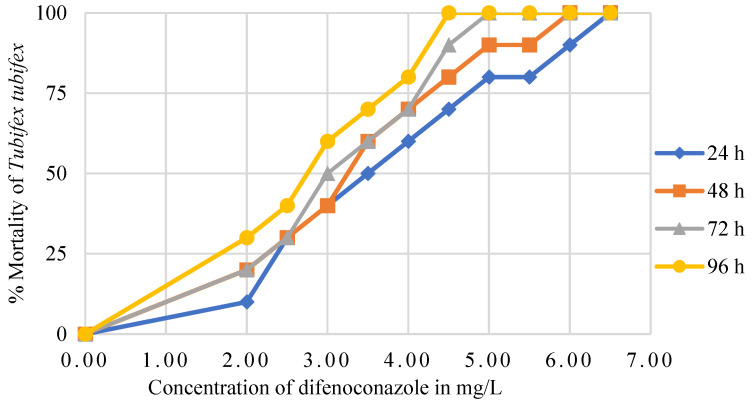
Mean percent mortality of *Tubifex tubifex* exposed to various concentrations of difenoconazole (2.00, 2.50, 3.00, 3.50, 4.00, 4.50, 5.50, 6.00, and 6.50 mg/L) over different exposure periods (24, 48, 72, and 96 h).

**Figure 2:**
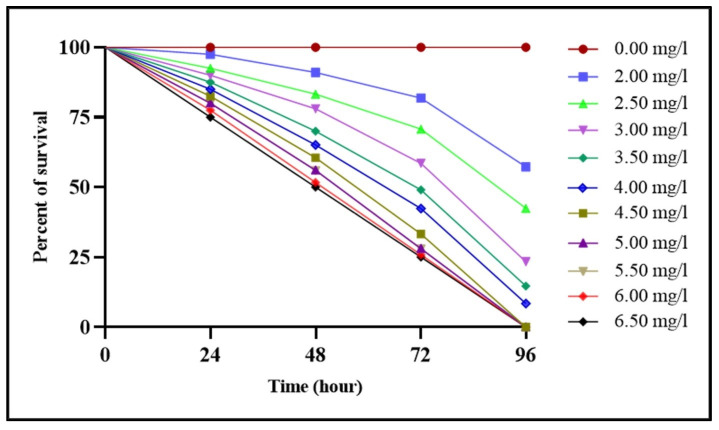
Kaplan–Meier survival curves for *Tubifex tubifex* exposed to different concentrations of difenoconazole, analyzed using the log-rank (Mantel–Cox) test (Chi-square = 116.7; df = 10; *p* < 0.0001).

The lethal concentrations (LC_10_, LC_20_, LC_30_, LC_40_, and LC_50_) of difenoconazole for *Tubifex tubifex* over a 96 h period, with 24 h intervals, are presented in Table 1, along with their 95% confidence intervals. No mortality was observed in the control group throughout the experiment. The mortality rate of the test animals was found to be significantly correlated with both the concentration of the toxicant and the duration of exposure, increasing in a concentration- and time-dependent manner (Figure 1). Additionally, the Kaplan–Meier survival curve indicates that difenoconazole significantly reduced the overall survival rates of the exposed test animals compared to the controls, with the effects dependent on both concentration and exposure duration (Mantel log-rank test; *p* < 0.05) (Figure 2).

In this study, the LC_50_ values of difenoconazole for *T. tubifex* at 24, 48, 72, and 96 h were determined to be 3.456, 3.094, 2.994, and 2.680 mg/L, respectively. These values are lower than the LC_50_ values reported for many other aquatic species [57,58,59,60]. Ahmad et al. reported that the 24 h LC_50_ value of difenoconazole observed for freshwater shrimp (*Macrobrachium lanchesteri*) was determined to be 2.91 mg/L [57]. The variations in LC_50_ values are likely due to differences in the size, age, and health condition of the test species, as well as variations in the physicochemical properties of the water [61,62,63]. Based on these comparisons, we conclude that *T. tubifex* is less resistant to difenoconazole than other aquatic invertebrates, as indicated by the lower LC_50_ values observed in this study compared to those reported in the literature.

#### 3.1.2. General Unified Threshold Model of Survival (GUTS) Analysis

The environmental effects of propiconazole were assessed by analyzing its toxicokinetic–toxicodynamic (TKTD) data at various concentrations and over time. To validate these findings, the General Unified Threshold Model for Survival (GUTS)-modified TKTD model was used.

**Figure 3:**
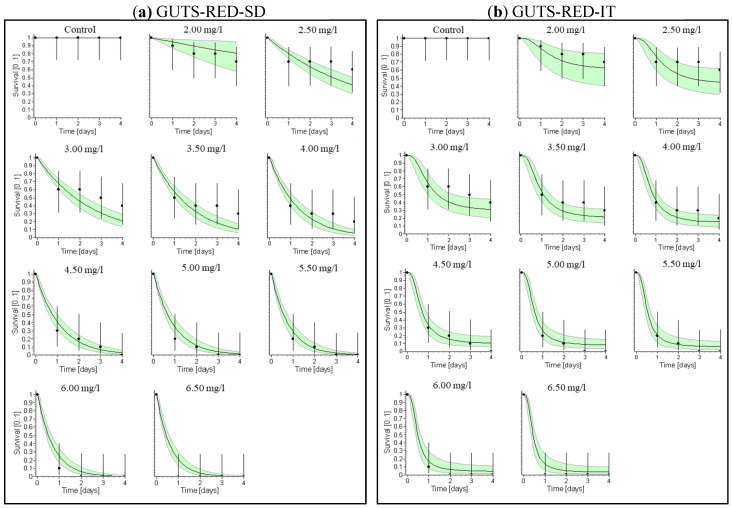
Relative fit of observed and fitted values of the (**a**) GUTS-SD and (**b**) GUTS-IT models at different difenoconazole exposure concentrations to *Tubifex tubifex*.

**Figure 4:**
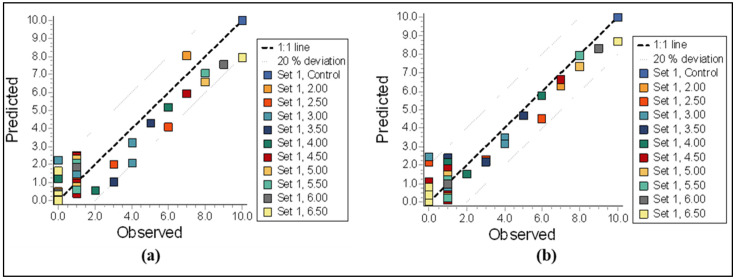
Comparison of the parameters estimated using the General Unified Threshold Model for Survival (GUTS); observed vs. predicted survival plot of propiconazole for the calibration of the (**a**) GUTS-RED-SD and (**b**) GUTS-RED-IT models.

In the GUTS-SD simulation analysis, the survival rate was accurately predicted at 0 mg/L for all levels of difenoconazole but was overestimated at 2.00 mg/L (Figure 3a). Similarly, in the GUTS-IT model simulation, the survival rates were accurately predicted at 0 mg/L for all concentrations of difenoconazole but were overestimated at 6.00 and 6.50 mg/L and underestimated at 2.00 mg/L (Figure 3b). The observed and predicted survival plots for difenoconazole are shown in Figure 4a,b. The GUTS-SD model demonstrated a better fit for these fungicides compared to the GUTS-IT model, as indicated by the Akaike Information Criterion (AIC) values (GUTS-SD = 203.66; GUTS-IT = 204.9). Lower AIC values suggest a better fit.

The GUTS analysis revealed that the GUTS-SD model is a more accurate predictor for the survival rate (LC_50_, LC_10_) of difenoconazole in *T. tubifex* than the GUTS-IT model (Table 2). The GUTS-SD model provided a reliable 4-day LC_50_ value of 2.351 mg/L, consistent with experimental LC_50_ values. Additionally, the model estimated the 100-day lethal concentration at 50% (LC_50_) for difenoconazole to be 1.86 mg/L (Table 2). Given the susceptibility of *T. tubifex* to difenoconazole, these findings are valuable for determining the Regulatory Acceptable Concentration (RAC) for aquatic environments exposed to organic acid pollutants over extended periods.

#### 3.1.3. Evaluation of the Toxicity Factors

Table 3 presents the toxicity factor values for *T. tubifex* exposed to difenoconazole over different durations (24, 48, 72, and 96 h).

The toxicity factor of difenoconazole for *T. tubifex* increased progressively with longer exposure times, from 24 to 96 h. Acute toxicity refers to the harmful effects of a substance resulting from either a single exposure or repeated exposures within a short period [64]. Acute toxicity assessments typically focus on dose-dependent adverse effects, with mortality being a primary endpoint [65]. A common method for evaluating toxicity is determining the LC_50_ value, which reflects the concentration at which 50% of the population is affected by the contaminant [66]. In this study, the toxicity factor (TF) was utilized to assess *T. tubifex*’s tolerance to difenoconazole at various exposure durations. Tolerance describes an organism’s ability to endure adverse environmental conditions, and calculating the toxicity factor helps quantify an organism’s sensitivity to a specific toxicant.

### 3.2. Sublethal Exposure Assessment

#### 3.2.1. Alterations in Oxidative Stress Enzyme

**Figure 5:**
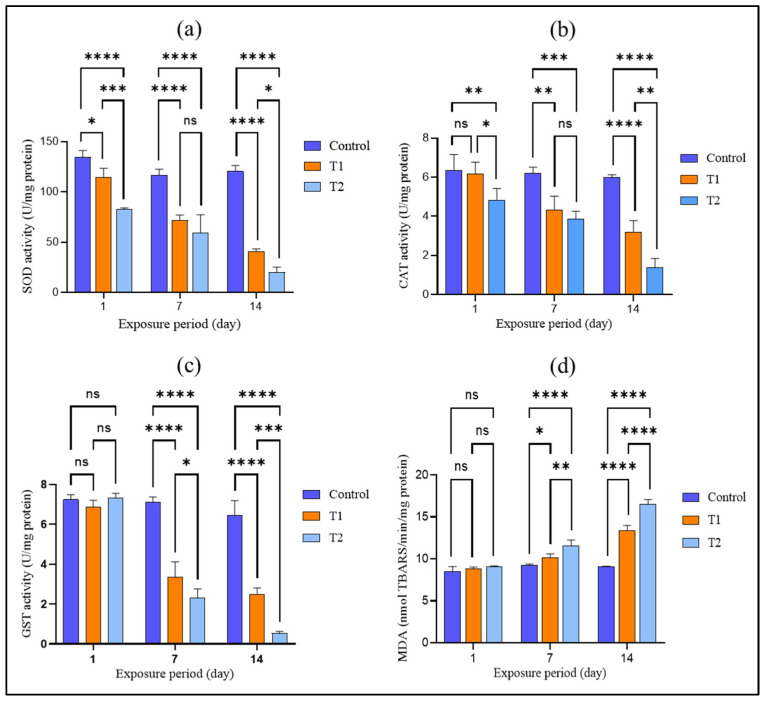
Effects of different sublethal concentrations of difenoconazole on (**a**) SOD, (**b**) CAT, (**c**) GST, and (**d**) MDA levels in *Tubifex tubifex* at different exposure periods. T1 and T2 correspond to difenoconazole concentrations at 10% and 20% of the 96 h LC_50_ value (0.268 mg/L and 0.536 mg/L, respectively). Statistical significance is indicated as follows: * *p* ≤ 0.05, ** *p* ≤ 0.01, *** *p* ≤ 0.001, **** *p* ≤ 0.0001; ns = not significant.

Antioxidant enzymes serve as key biomarkers of oxidative stress by neutralizing reactive oxygen species (ROS) and certain pro-oxidative peptides under normal conditions. Tests involving *T. tubifex* exposed to difenoconazole were compared with control groups to evaluate oxidative stress responses. An analysis of these enzymes and MDA levels revealed significant changes: the CAT, SOD, and GST activities were notably suppressed, while the MDA concentration increased significantly (*p* < 0.05) in the difenoconazole-treated groups, as illustrated in Figure 5a–d. These findings are consistent with previous research [67,68].

SOD is a critical enzyme that protects cells from oxidative stress by converting reactive oxygen radicals into hydrogen peroxide (H_2_O_2_) through dismutation [69,70,71,72]. Our study observed a significant reduction in the SOD activity—15% and 38% on day 1 and 39% and 49% on day 7—at difenoconazole concentrations of 0.268 mg/L and 0.536 mg/L, respectively, compared to the control. On day 14, the SOD activity further decreased in both exposed groups. This decreases likely resulted from the generation of excess ROS, which oxidize cysteine residues in SOD, impairing its function or reducing its gene expression [73,74].

CAT functions as a crucial scavenger of hydrogen peroxide (H_2_O_2_), protecting cells from oxidative damage by mitigating oxygen radical toxicity [75]. In *T. tubifex* exposed to difenoconazole, the CAT activity was significantly reduced (*p* < 0.05). On day 1, the CAT activity was notably lower in the T2 group compared to the T1 group and the control. By day 7, the CAT activity decreased by 30% and 38%, and by day 14, it had decreased by 47% and 77% in the T1 and T2 groups, respectively. The reduction in CAT activity is likely due to peroxidative damage to essential cellular components and/or increased ROS production, similar to the findings in freshwater fish exposed to amino-triazole [76].

GST is a major biotransformation enzyme in phase II detoxification that facilitates the accumulation of glutathione (GSH) and detoxifies xenobiotics [77]. In our study, GST activity showed a minor increase in the T2 group but a slight decrease in the T1 group on day 1 compared to controls. The GST activity significantly declined by 53% and 67% on day 7 and further decreased by 62% and 92% on day 14 in the T1 and T2 groups, respectively. This sharp decline may be due to the downregulation of GST-related genes, leading to decreased enzyme activity [78]. The downregulation of GST genes can prevent nuclear transcription factors from binding to their promoter regions, resulting in excessive ROS production [79]. Previous studies have also shown that difenoconazole can reduce SOD, CAT, and GST mRNA expression in zebrafish [80], indicating that oxidative stress plays a role in difenoconazole-induced damage in *T. tubifex*. Similar GST level changes have been observed in *Tubifex* exposed to chitosan [81].

Oxidative stress leads to reactions between ROS and unsaturated fatty acids in cell membranes, resulting in lipid peroxidation (LPO). Increased LPO promotes further ROS generation [82,83]. LPO can be assessed via MDA levels, a sensitive marker of oxidative cell damage [84]. Elevated MDA levels were observed in both difenoconazole-exposed groups (T1 and T2) in a concentration- and time-dependent manner (Figure 5d), indicating enhanced ROS production [85]. Difenoconazole may increase MDA levels by interacting with polyunsaturated fatty acids under conditions of antioxidant enzyme deficiency, such as decreased GST activity [86]. Increased MDA levels can compromise cell membrane integrity, allowing toxic substances to enter and potentially cause DNA damage and apoptosis [87]. Similar MDA level increases were reported in Tubifex exposed to thallium for 15 days [88].

After 1 day of exposure, both GST and MDA did not differ (control vs. treatment organisms). The results indicated that the addition of difenoconazole increased the generation of reactive oxygen species (ROS). However, excessive ROS production impaired the effectiveness of these antioxidant enzymes, leading to the accumulation of malondialdehyde (MDA), mitochondrial dysfunction, and DNA damage [87]. Additionally, a two-way ANOVA analysis demonstrated that both the concentration of difenoconazole and the duration of exposure, as well as their interactions, significantly affected all oxidative stress biomarkers studied (SOD, CAT, GST, and MDA).

#### 3.2.2. Integrated Biomarker Response

**Figure 6:**
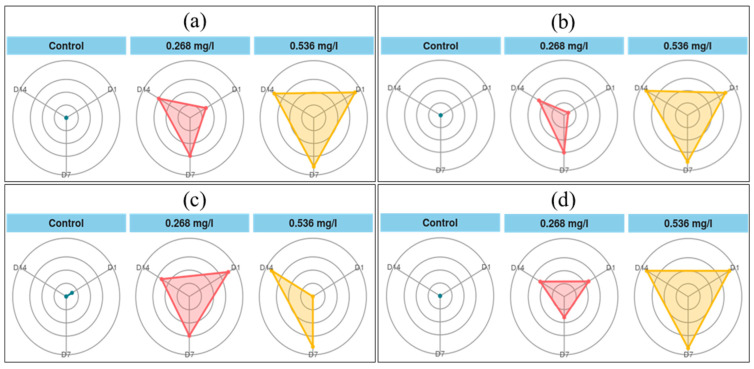
IBR star plots for analyzing (**a**)-SOD, (**b**)-CAT, (**c**)-MDA, and (**d**)-GST biomarker responses in Tubifex tubifex exposed to different difenoconazole concentrations across varied exposure duration (days).

The Integrated Biomarker Response (IBR) index was employed to assess the overall stress caused by difenoconazole on *Tubifex tubifex* (Figure 6). The IBR is a robust method for consolidating multiple biomarkers into a single numerical value, providing a comprehensive measure of stress [54,89]. Generally, a high IBR indicates poor ecological conditions for the organism, whereas a low IBR suggests healthier ecological conditions [1,6,90].

As shown in Table 4, control groups exhibited lower IBR values for SOD, CAT, MDA, and GST compared to the T1 and T2 treatment groups. Stress enzymes such as catalase (CAT), superoxide dismutase (SOD), and glutathione S-transferase (GST) are commonly used as biomarkers in toxicological studies [13]. However, relying on a single biomarker provides limited insight into the overall toxicity effects. Therefore, the IBR, which integrates multiple biomarkers, offers a more effective approach for interpreting the responses of organisms to toxicants [91,92,93]. Consequently, the toxicity of difenoconazole is more accurately assessed through oxidative stress biomarkers using IBR values.

#### 3.2.3. Correlation Analysis

**Figure 7:**
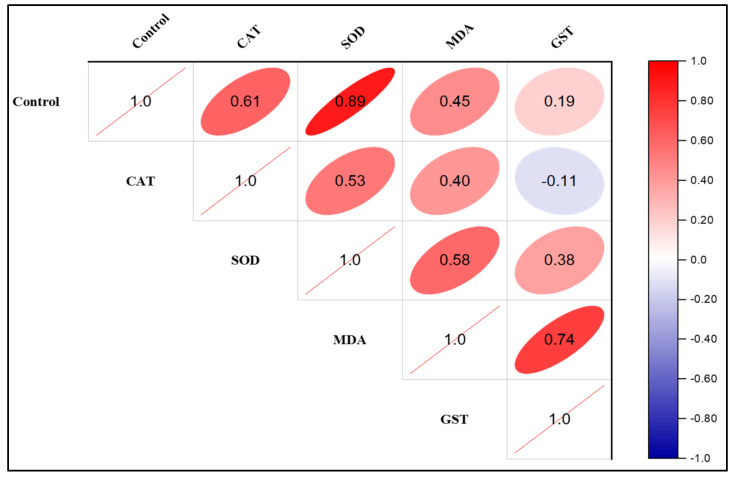
Correlation plot between CAT, SOD, MDA, and GST after difenoconazole exposure to *T. tubifex*.

Using oxidative stress biomarkers such as catalase (CAT), superoxide dismutase (SOD), malondialdehyde (MDA), and glutathione S-transferase (GST), the Pearson correlation matrix displays the significance level between difenoconazole exposure (mg/L) and the exposure duration in comparison with the control (Figure 7). The study found that as the level of difenoconazole increased, the MDA level in the worms increased.

#### 3.2.4. Histological Alterations

**Figure 8:**
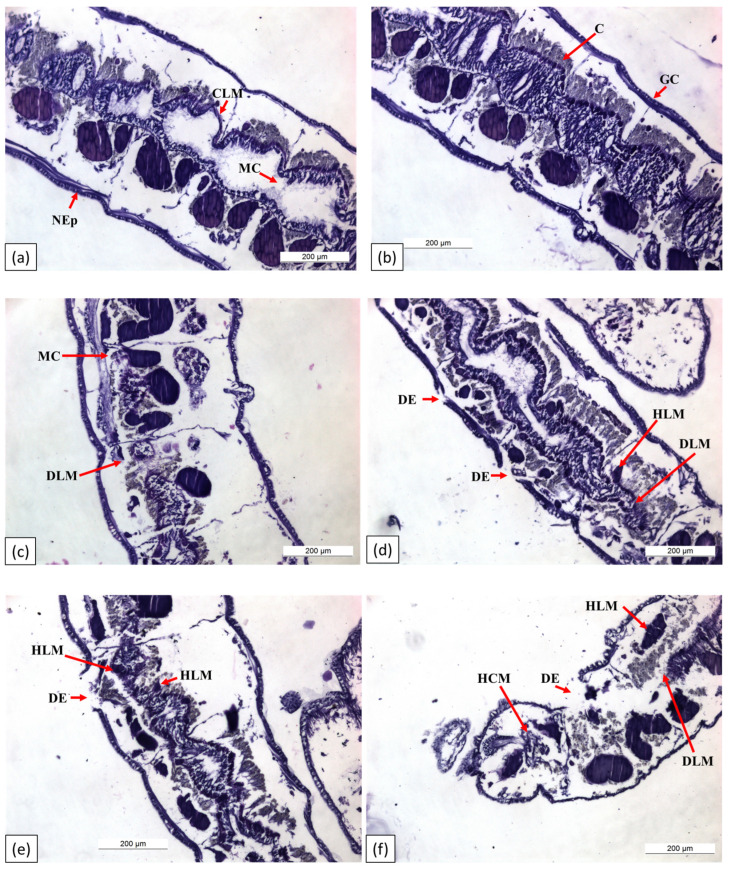
Longitudinal section (H&E) of Tubifex tubifex. (**a**,**b**) Control: normal epidermis (NEp), glandular cells of the epidermis (GCs) and totally stretched continuity of the longitudinal muscle (CLM), chloragogenous cells (Cs), and mucus channel (MC) of worm; (**c**,**d**) treated with 0.268 mg/L difenoconazole: disintegrating epidermis (DE), extensive disintegration of longitudinal muscles (DLMs) and mucus channels (MCs) in segments, and hypertrophied remnants of longitudinal muscles (HLMs); (**e**,**f**) treated with 0.536 mg/L difenoconazole: disintegrated longitudinal muscles (DLMs), hypertrophied longitudinal muscles (HLMs), hypertrophied circular muscle (HCM), and disintegration of the epidermis (DE).

Longitudinal histological sections of the test worms revealed an outer layer of epithelial cells, or epidermis, that appeared continuous with the integumental surface (Figure 8). In the control group, the glandular cells of the epidermis (GC), epidermis (NEp), and longitudinal muscle (CLM) showed a normal appearance, with intact chloragogenous cells (Cs) and mucus channels (MCs) (Figure 8a,b).

In worms treated with 0.268 mg/L difenoconazole (Figure 8c,d), notable features included extensive disintegration of the epidermis (DE), severe disintegration of the longitudinal muscles (DLMs) and mucus channels (MCs) in various segments, and hypertrophied remnants of longitudinal muscles (HLMs). In worms exposed to 0.536 mg/L difenoconazole (Figure 8e,f), the primary pathological changes included extensive disintegration of longitudinal muscles (DLMs), hypertrophied longitudinal muscles (HLMs), hypertrophied circular muscles (HCMs), and disintegration of the epidermis (DE).

The photomicrographs (Figure 8a–f) show that worms treated with 10% and 20% difenoconazole exhibited a significant loss of longitudinal muscles and pronounced thinning and degeneration of the epithelial cells. The changes in the alimentary canal cells observed in the exposed groups were often dependent on the concentration and toxicity of the difenoconazole. Different concentrations of difenoconazole altered the shape of the epithelial cells, particularly in the digestive tract.

## 4. Conclusions

Pesticides are widely used in agriculture to protect crops from pests and diseases, but they can also enter nearby aquatic environments through natural processes. Factors like weather patterns, such as rainfall, and the physicochemical properties of the pesticides, such as hydrophobicity and stability, influence their distribution and persistence in water. Human activities, including spills, urban runoff, and improper disposal, further impact pesticide concentrations in aquatic systems. This study examines the effects of the fungicide difenoconazole on the tubificid worm *Tubifex tubifex*. The research shows that *T. tubifex* is particularly sensitive to difenoconazole compared to other aquatic invertebrates. Exposure led to increased ROS, inhibited antioxidant enzyme activity, and caused MDA formation. Significant histological changes were also observed with treatments of 10% and 20% of the 96 h LC_50_. These findings underscore the need for better understanding of pesticide impacts on aquatic life and help establish Regulatory Acceptable Concentrations (RACs).

## Figures and Tables

**Table 1:** Lethal concentrations (LC_10_, LC_20_, LC_30_, LC_40_, and LC_50_) with 95% fiducial limits of difenoconazole to *Tubifex tubifex* at exposure times of 24, 48, 72, and 96 h.

Lethal Concentration		Concentration with 95% Confidence Intervals (mg/L)			
		24 h	48 h	72 h	96 h
*Tubifex tubifex*	LC_10_	1.884 [1.653–2.146]	1.755 [1.545–1.994]	1.752 [1.549–1.983]	1.421 [1.227–1.645]
	LC_20_	2.320 [2.036–2.643]	2.132 [1.876–2.422]	2.106 [1.862–2.383]	1.767 [1.525–2.046]
	LC_30_	2.696 [2.367–3.071]	2.453 [2.159–2.787]	2.405 [2.125–2.721]	2.067 [1.785–2.394]
	LC_40_	3.065 [2.691–3.492]	2.766 [2.434–3.142]	2.693 [2.380–3.047]	2.364 [2.041–2.738]
	LC_50_	3.456 [3.034–3.937]	3.094 [2.723–3.515]	2.994 [2.646–3.387]	2.680 [2.314–3.104]

**Table 2:** Predicted LC_50_ and LC_10_ values (mg/L) of difenoconazole using GUTS-SD modeling for 1, 2, 3, 4, 7, 21, 50, and 100 days with 95% confidence intervals (CIs).

Time (Day)	LC_50_ (95% CI)	LC_10_ (95% CI)
1	3.900 (3.473–4.508)	2.155 (1.895–2.291)
2	2.865 (2.626–3.153)	1.996 (1.708–2.118)
3	2.522 (2.294–2.726)	1.943 (1.644–2.064)
4	2.351 (2.113–2.519)	1.917 (1.611–2.037)
7	2.131 (1.868–2.262)	1.884 (1.570–2.003)
21	1.936 (1.635–2.057)	1.854 (1.533–1.975)
50	1.880 (1.565–2.000)	1.845 (1.523–1.967)
100	1.860 (1.540–1.981)	1.842 (1.519–1.964)

**Table 3:** Toxicity factors of difenoconazole at 24, 48, 72, and 96 h exposures to *Tubifex tubifex*.

Test Animal	Toxicity Factor (TF)			
	24 h	48 h	72 h	96 h
*Tubifex tubifex*	1.000	1.117	1.154	1.289

**Table 4:** IBR values of different biomarkers to different difenoconazole exposure concentrations.

Biomarker	Control	T1 (0.268 mg/L)	T2 (0.536 mg/L)
SOD	0.00	1.95	4.82
CAT	0.00	1.03	4.69
GST	0.00	3.10	1.62
MDA	0.00	1.39	5.14

## Data Availability

All data are included in this article.

## Supplementary Materials

The following supporting information can be downloaded at: https://www.mdpi.com/article/10.3390/biology14030302/s1: Table S1: Raw data for HPLC chromatogram; Figure S1: HPLC chromatogram illustrating the stability of DIF concentration in test water. No significant dissipation of DIF was observed.
