# Supplementary material for: Effects of Difenoconazole on Tubifex tubifex: Antioxidant Activity, Insights from GUTS Predictions, and Multi-Biomarker Analysis
Source: Biology (Basel). 2025 Mar 17;14(3):302. doi: 10.3390/biology14030302 (PMC11939907; doi:10.3390/biology14030302)
Supplement: Supplementary file 1 [file biology-14-00302-s001.zip › biology-3350730-supplementary.pdf]

Supplementary materials

Table S1. Raw data for HPLC chromatogram

Day 0		Day 4	
t	Ct	t	Ct
0.00	0.00	0.00	0.00
0.04	0.00	0.04	0.00
0.07	0.00	0.07	0.00
0.10	0.00	0.10	0.00
0.13	0.00	0.13	0.00
0.17	0.00	0.17	0.00
0.20	0.00	0.20	0.00
0.23	0.00	0.23	0.00
0.27	0.00	0.27	0.00
0.30	0.00	0.30	0.00
0.33	0.00	0.33	0.00
0.36	0.00	0.36	0.00
0.40	0.00	0.40	0.00
0.43	0.00	0.43	0.00
0.46	0.00	0.46	0.00
0.50	0.00	0.50	0.00
0.53	0.00	0.53	0.00
0.56	0.00	0.56	0.00
0.59	0.00	0.59	0.00
0.63	0.00	0.63	0.00
0.66	0.00	0.66	0.00
0.69	0.00	0.69	0.00
0.73	0.00	0.73	0.00
0.76	0.00	0.76	0.00
0.79	0.00	0.79	0.00
0.82	0.00	0.82	0.00
0.86	0.00	0.86	0.00
0.89	0.00	0.89	0.00
0.92	0.00	0.92	0.00
0.96	0.00	0.96	0.00
0.99	0.00	0.99	0.00
1.02	0.00	1.02	0.00
1.05	0.00	1.05	0.00
1.09	0.00	1.09	0.00
1.12	0.00	1.12	0.00
1.15	0.00	1.15	0.00
1.19	0.00	1.19	0.00
1.22	0.00	1.22	0.00
1.25	0.00	1.25	0.00
1.28	0.00	1.28	0.00
1.32	0.00	1.32	0.00
1.35	0.00	1.35	0.00
1.38	0.00	1.38	0.00
1.42	0.00	1.42	0.00

1.45	0.00	1.45	0.00
1.48	0.00	1.48	0.00
1.51	0.00	1.51	0.00
1.55	0.00	1.55	0.00
1.58	0.00	1.58	0.00
1.61	0.00	1.61	0.00
1.65	0.00	1.65	0.00
1.68	0.00	1.68	0.00
1.71	0.00	1.71	0.00
1.74	0.00	1.74	0.00
1.78	0.00	1.78	0.00
1.81	0.00	1.81	0.00
1.84	0.00	1.84	0.00
1.88	0.00	1.88	0.00
1.91	0.00	1.91	0.00
1.94	0.00	1.94	0.00
1.97	0.00	1.97	0.00
2.01	0.00	2.01	0.00
2.04	0.00	2.04	0.00
2.07	0.00	2.07	0.00
2.11	0.00	2.11	0.00
2.14	0.00	2.14	0.00
2.17	0.00	2.17	0.00
2.20	0.00	2.20	0.00
2.24	0.00	2.24	0.00
2.27	0.00	2.27	0.00
2.30	0.00	2.30	0.00
2.34	0.00	2.34	0.00
2.37	0.00	2.37	0.00
2.40	0.00	2.40	0.00
2.43	0.00	2.43	0.00
2.47	0.00	2.47	0.00
2.50	0.00	2.50	0.00
2.53	0.00	2.53	0.00
2.57	0.00	2.57	0.00
2.60	0.00	2.60	0.00
2.63	0.00	2.63	0.00
2.66	0.00	2.66	0.00
2.70	0.00	2.70	0.00
2.73	0.00	2.73	0.00
2.76	0.00	2.76	0.00
2.80	0.00	2.80	0.00
2.83	0.00	2.83	0.00
2.86	0.00	2.86	0.00
2.89	0.00	2.89	0.00
2.93	0.00	2.93	0.00
2.96	0.00	2.96	0.00
2.99	0.00	2.99	0.00

3.03	0.00	3.03	0.00
3.06	0.00	3.06	0.00
3.09	0.00	3.09	0.00
3.12	0.00	3.12	0.00
3.16	0.00	3.16	0.00
3.19	0.00	3.19	0.00
3.22	0.00	3.22	0.00
3.26	0.00	3.26	0.00
3.28	0.00	3.28	0.00
3.32	0.00	3.32	0.00
3.35	0.00	3.35	0.00
3.38	0.00	3.38	0.00
3.41	0.00	3.41	0.00
3.45	0.00	3.45	0.00
3.48	0.00	3.48	0.00
3.51	0.00	3.51	0.00
3.54	0.19	3.54	0.19
3.57	0.00	3.57	0.00
3.61	0.64	3.61	0.64
3.64	0.00	3.64	0.00
3.67	0.00	3.67	0.00
3.70	0.00	3.70	0.00
3.74	0.00	3.74	0.00
3.77	0.00	3.77	0.00
3.80	0.00	3.80	0.00
3.83	0.00	3.83	0.00
3.86	0.00	3.86	0.00
3.90	0.00	3.90	0.00
3.93	0.00	3.93	0.00
3.96	0.13	3.96	0.34
3.99	0.00	3.99	0.00
4.03	0.00	4.03	0.00
4.06	0.00	4.06	0.00
4.09	0.00	4.09	0.00
4.12	0.00	4.12	0.00
4.15	0.00	4.15	0.00
4.19	0.00	4.19	0.00
4.22	0.00	4.22	0.00
4.25	0.00	4.25	0.00
4.28	0.00	4.28	0.00
4.32	0.00	4.32	0.00
4.35	0.00	4.35	0.00
4.38	0.00	4.38	0.00
4.41	0.00	4.41	0.00
4.45	0.00	4.45	0.00
4.48	0.00	4.48	0.00
4.51	0.00	4.51	0.00
4.54	0.00	4.54	0.00

4.57	0.00	4.57	0.00
4.61	0.00	4.61	0.00
4.64	0.00	4.64	0.00
4.67	0.00	4.67	0.00
4.70	0.00	4.70	0.00
4.74	0.00	4.74	0.00
4.77	0.00	4.77	0.00
4.80	0.00	4.80	0.00
4.83	0.00	4.83	0.00
4.86	0.00	4.86	0.00
4.90	0.00	4.90	0.00
4.93	0.00	4.93	0.00
4.96	0.00	4.96	0.00
4.99	0.00	4.99	0.00
5.03	0.00	5.03	0.00
5.06	1.01	5.06	1.01
5.09	0.00	5.09	0.00
5.12	0.00	5.12	0.00
5.15	0.00	5.15	0.00
5.19	0.00	5.19	0.00
5.22	0.00	5.22	0.00
5.25	0.00	5.25	0.00
5.28	0.00	5.28	0.00
5.32	0.00	5.32	0.00
5.35	0.00	5.35	0.00
5.38	0.00	5.38	0.00
5.41	0.00	5.41	0.00
5.45	0.00	5.45	0.00
5.48	0.00	5.48	0.00
5.51	0.00	5.51	0.00
5.54	0.00	5.54	0.00
5.57	0.00	5.57	0.00
5.61	0.01	5.61	0.01
5.64	0.00	5.64	0.00
5.67	0.00	5.67	0.00
5.70	0.00	5.70	0.00
5.74	0.00	5.74	0.00
5.77	0.00	5.77	0.00
5.80	0.00	5.80	0.00
5.83	0.00	5.83	0.00
5.86	0.00	5.86	0.00
5.90	0.00	5.90	0.00
5.93	1.01	5.93	1.01
5.96	0.00	5.96	0.00
5.99	0.00	5.99	0.00
6.03	0.00	6.03	0.00
6.06	0.00	6.06	0.00
6.09	0.00	6.09	0.00

6.12	0.00	6.12	0.00
6.15	0.00	6.15	0.00
6.19	0.00	6.19	0.00
6.22	0.00	6.22	0.00
6.25	0.00	6.25	0.00
6.28	0.00	6.28	0.00
6.32	0.00	6.32	0.00
6.35	0.00	6.35	0.00
6.38	0.00	6.38	0.00
6.41	0.00	6.41	0.00
6.45	0.00	6.45	0.00
6.48	0.00	6.48	0.00
6.51	0.00	6.51	0.00
6.54	0.00	6.54	0.00
6.57	0.00	6.57	0.00
6.61	0.00	6.61	0.00
6.64	0.00	6.64	0.00
6.67	0.00	6.67	0.00
6.70	0.00	6.70	0.00
6.74	0.00	6.74	0.00
6.77	0.00	6.77	0.00
6.80	0.00	6.80	0.00
6.83	0.00	6.83	0.00
6.86	0.00	6.86	0.00
6.90	0.00	6.90	0.00
6.93	0.00	6.93	0.00
6.96	0.00	6.96	0.00
6.99	0.00	6.99	0.00
7.03	0.00	7.03	0.00
7.06	0.00	7.06	0.00
7.09	0.00	7.09	0.00
7.12	0.00	7.12	0.00
7.15	0.00	7.15	0.00
7.19	0.00	7.19	0.00
7.22	0.00	7.22	0.00
7.25	0.00	7.25	0.00
7.28	0.00	7.28	0.00
7.32	0.00	7.32	0.00
7.35	0.00	7.35	0.00
7.38	0.00	7.38	0.00
7.41	0.00	7.41	0.00
7.45	0.00	7.45	0.00
7.48	0.00	7.48	0.00
7.51	0.00	7.51	0.00
7.54	0.01	7.54	0.01
7.57	0.00	7.57	0.00
7.61	0.00	7.61	0.00
7.64	0.00	7.64	0.00

Supplementary materials

7.67	0.00	7.67	0.00
7.70	0.00	7.70	0.00
7.74	0.00	7.74	0.00
7.77	0.00	7.77	0.00
7.80	0.00	7.80	0.00
7.83	0.00	7.83	0.00
7.86	0.00	7.86	0.00
7.90	0.00	7.90	0.00
7.93	0.00	7.93	0.00
7.96	0.00	7.96	0.00
7.99	0.00	7.99	0.00
8.03	0.00	8.03	0.00
8.06	0.00	8.06	0.00
8.09	0.00	8.09	0.00
8.12	0.00	8.12	0.00
8.15	0.00	8.15	0.00
8.19	0.00	8.19	0.00
8.22	0.00	8.22	0.00
8.25	0.00	8.25	0.00
8.28	0.00	8.28	0.00
8.32	0.00	8.32	0.00
8.35	0.00	8.35	0.00
8.38	0.00	8.38	0.00
8.41	0.00	8.41	0.00
8.45	0.00	8.45	0.00
8.48	0.00	8.48	0.00
8.51	0.00	8.51	0.00
8.54	0.00	8.54	0.00
8.57	0.00	8.57	0.00
8.61	0.00	8.61	0.00
8.64	0.00	8.64	0.00
8.67	0.00	8.67	0.00
8.70	0.00	8.70	0.00
8.74	0.00	8.74	0.00
8.77	0.00	8.77	0.00
8.80	0.00	8.80	0.00
8.83	0.00	8.83	0.00
8.86	0.00	8.86	0.00
8.90	0.00	8.90	0.00
8.93	0.00	8.93	0.00
8.96	0.00	8.96	0.00
8.99	0.00	8.99	0.00
9.03	0.00	9.03	0.00
9.06	0.00	9.06	0.00
9.09	0.00	9.09	0.00
9.12	0.00	9.12	0.00
9.15	0.00	9.15	0.00
9.19	0.00	9.19	0.00

9.22	0.00	9.22	0.00
9.25	1.01	9.25	1.01
9.28	0.00	9.28	0.00
9.32	0.00	9.32	0.00
9.35	0.00	9.35	0.00
9.38	0.00	9.38	0.00
9.41	0.00	9.41	0.00
9.45	0.00	9.45	0.00
9.48	0.00	9.48	0.00
9.51	0.00	9.51	0.00
9.54	0.00	9.54	0.00
9.57	0.00	9.57	0.00
9.61	0.00	9.61	0.00
9.64	0.00	9.64	0.00
9.67	0.00	9.67	0.00
9.70	0.00	9.70	0.00
9.74	0.00	9.74	0.00
9.77	0.00	9.77	0.00
9.80	0.00	9.80	0.00
9.83	0.00	9.83	0.00
9.86	0.00	9.86	0.00
9.90	0.12	9.90	0.96
9.93	0.00	9.93	0.00
9.96	0.00	9.96	0.00
9.99	0.00	9.99	0.00
10.03	0.00	10.03	0.00
10.06	0.00	10.06	0.00
10.09	0.00	10.09	0.00
10.12	0.00	10.12	0.00
10.15	0.00	10.15	0.00
10.19	0.00	10.19	0.00
10.22	0.00	10.22	0.00
10.25	0.00	10.25	0.00
10.28	0.00	10.28	0.00
10.32	0.00	10.32	0.00
10.35	0.00	10.35	0.00
10.38	0.00	10.38	0.00
10.41	0.00	10.41	0.00
10.45	0.00	10.45	0.00
10.48	0.00	10.48	0.00
10.51	0.00	10.51	0.00
10.54	0.00	10.54	0.00
10.57	0.00	10.57	0.00
10.61	0.00	10.61	0.00
10.64	0.00	10.64	0.00
10.67	0.00	10.67	0.00
10.70	0.00	10.70	0.00
10.74	0.00	10.74	0.00

Supplementary materials

10.77	0.00	10.77	0.00
10.80	0.00	10.80	0.00
10.83	0.00	10.83	0.00
10.86	0.00	10.86	0.00
10.90	0.02	10.90	0.02
10.93	0.12	10.93	0.11
10.96	0.58	10.96	0.55
10.99	2.37	10.99	2.27
11.03	8.09	11.03	7.77
11.06	23.02	11.06	22.10
11.09	54.52	11.09	52.34
11.12	107.43	11.12	103.14
11.15	176.16	11.15	169.12
11.19	240.38	11.19	230.77
11.22	303.16	11.22	291.04
11.25	257.93	11.25	242.45
11.28	202.82	11.28	190.65
11.32	132.72	11.32	124.75
11.35	72.27	11.35	67.93
11.38	32.75	11.38	30.78
11.41	12.35	11.41	11.61
11.45	3.87	11.45	3.64
11.48	1.01	11.48	0.95
11.51	0.22	11.51	0.21
11.54	0.04	11.54	3.62
11.57	0.01	11.57	0.54
11.61	0.00	11.61	0.07
11.64	0.00	11.64	0.01
11.67	0.00	11.67	0.00
11.70	0.00	11.70	0.00
11.74	0.00	11.74	0.00
11.77	0.00	11.77	0.00
11.80	0.00	11.80	0.00
11.83	0.00	11.83	0.00
11.86	2.00	11.86	1.00
11.90	0.00	11.90	0.00
11.93	0.00	11.93	0.00
11.96	0.00	11.96	0.00
11.99	0.00	11.99	0.00
12.03	0.00	12.03	0.00

Supplementary materials

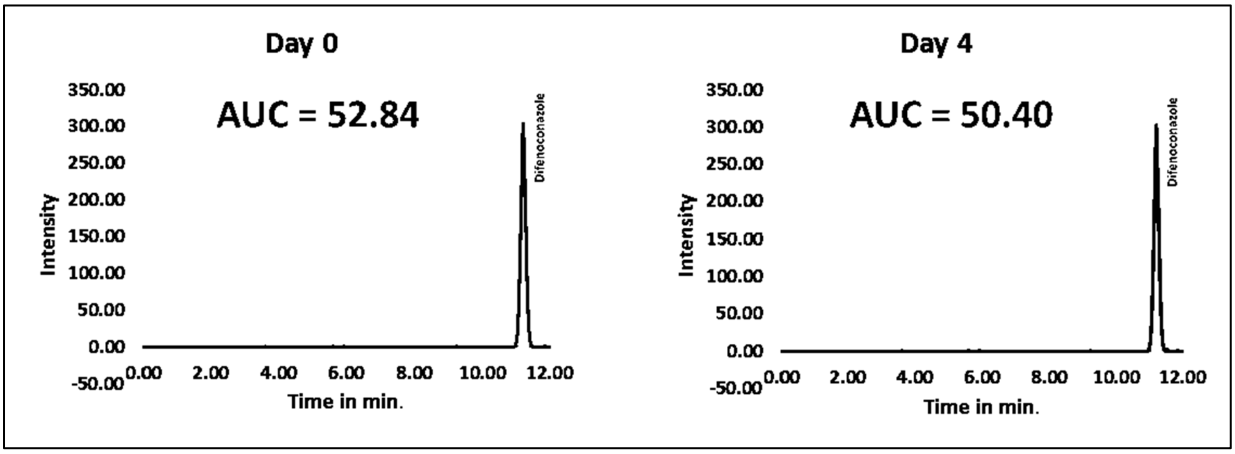

Figure S1. HPLC chromatogram illustrating the stability of DIF concentration in test water. No significant dissipation of DIF was observed
